# Topographic Organization of Cholinergic Innervation From the Basal Forebrain to the Visual Cortex in the Rat

**DOI:** 10.3389/fncir.2018.00019

**Published:** 2018-03-08

**Authors:** Frédéric Huppé-Gourgues, Karim Jegouic, Elvire Vaucher

**Affiliations:** ^1^Laboratoire de Neurobiologie de la Cognition Visuelle, École d’Optométrie, Université de Montréal, Montreal, QC, Canada; ^2^École de Psychologie, Université de Moncton, Moncton, NB, Canada

**Keywords:** cholera toxin B, visual cortex, diagonal band of Broca, acetylcholine, topographic maps, Long–Evans rat, cortical neuromodulation

## Abstract

Acetylcholine is an important neurotransmitter for the regulation of visual attention, plasticity, and perceptual learning. It is released in the visual cortex predominantly by cholinergic projections from the basal forebrain, where stimulation may produce potentiation of visual processes. However, little is known about the fine organization of these corticopetal projections, such as whether basal forebrain neurons projecting to the primary and secondary visual cortical areas (V1 and V2, respectively) are organized retinotopically. The aim of this study was to map these basal forebrain-V1/V2 projections. Microinjections of the fluorescent retrograde tracer cholera toxin b fragment in different sites within V1 and V2 in Long–Evans rats were performed. Retrogradely labeled cell bodies in the horizontal and vertical limbs of the diagonal band of Broca (HDB and VDB, respectively), nucleus basalis magnocellularis, and substantia innominata (SI), were mapped *ex vivo* with a computer-assisted microscope stage controlled by stereological software. Choline acetyltranferase immunohistochemistry was used to identify cholinergic cells. Our results showed a predominance of cholinergic projections coming from the HDB. These projections were not retinotopically organized but projections to V1 arised from neurons located in the anterior HDB/SI whereas projections to V2 arised from neurons located throughout the whole extent of HDB/SI. The absence of a clear topography of these projections suggests that BF activation can stimulate visual cortices broadly.

## Introduction

The neurotransmitter acetylcholine (ACh) is important for the tuning of cortical functions through mechanisms that modulate synaptic strength and neural responses. ACh can produce a broad network-coordinating signal that elevates cortical activity and can also produce localized signals that modulate the neural activity of specific cells (Sarter et al., 2005; Hasselmo and Sarter, 2011). These two signaling forms echo to the underlying organization of cholinergic innervation. On one hand, cholinergic innervation of the cortex consists of topographically organized projections from cells in the basal forebrain (BF) (Rye et al., 1984; Luiten et al., 1987; Gaykema et al., 1990; Woolf, 1991; Vaucher and Hamel, 1995; Zaborszky et al., 2015). ACh is contained and most probably released from varicosities distributed along these long cholinergic axons, suggesting a broad ACh role. The low synaptic frequency on cholinergic varicosities demonstrated by ultrastructural studies (Vaucher and Hamel, 1995; Mechawar et al., 2000) also fits with broad volume transmission rather than restricted synaptic transmission (Descarries and Mechawar, 2000; Yamasaki et al., 2010). On the other hand, ACh may exert localized micro-function effects within specific cortical areas or layers owing to the enriched distribution of varicosities along specific segments of cholinergic axons (Zhang et al., 2011) or of cholinergic receptor subtypes (Hasselmo and Sarter, 2011; Coppola et al., 2016). In addition, restricted cholinergic actions could be generated by locally evoked ACh release. It is not yet known whether cholinergic projections are distributed differentially according to higher cortical functional organization, such as retinotopy in the visual cortex.

Local cholinergic modulation of visual cortical areas has been evidenced in a number of rat studies (Fournier et al., 2004; Laplante et al., 2005; Soma et al., 2013). ACh has been shown to play a role in visual attention (Sarter et al., 2005; Herrero et al., 2008), as well as in the refining of visual perception (Kang et al., 2014; Gratton et al., 2017) and visual cortex pathways (Ricciardi et al., 2013). Indeed, ACh influences the intensity of neural activity (Gil et al., 1997; Pinto et al., 2013; Soma et al., 2013), preferred responses (Roberts et al., 2005) and receptive field properties (Herrero et al., 2008) in primary visual cortex (V1). The presence of cholinergic terminals throughout the cortical layers of visual areas (Mechawar et al., 2000) suggests that ACh might affect visual processing at each level of visual processing. Visual areas are organized in a precise retinotopic manner, as shown in V1 in electrophysiological experiments (Adams and Forrester, 1968) and in the extra striate area (Montero et al., 1973; Espinoza and Thomas, 1983) in optical imaging experiments (Gias et al., 2005). Functional maps produced in these studies fit well with the cytoarchitectonic organization of visual cortex, as shown in the work of Krieg (1946), Paxinos (1995), and Paxinos and Watson (1997), wherein cellular densities delineate V1 and secondary visual cortex (V2) organization.

The BF origin of corticopetal ACh fibers has been described by Shute and Lewis (1967) and Mesulam et al. (1983). The cholinergic BF nuclei include the medial septum/vertical limb of the diagonal band of Broca (VDB), horizontal limb of the diagonal band of Broca (HDB), sublenticular substantia innominata (SI), and nucleus basalis magnocellularis (NBM). These areas contain heterogeneous populations of neurons, where cholinergic and non-cholinergic projection neurons are intermingled with putative interneurons along ascending pathways (Zaborszky and Duque, 2000). The NBM and SI innervate frontal regions (Rye et al., 1984; Luiten et al., 1987), whereas the HDB, VDB, and anterior BF project to visual cortex areas (Rye et al., 1984; Saper, 1984; Gaykema et al., 1990; Laplante et al., 2005). The SI projects, but only weakly, to V1 (Rye et al., 1984; Vaucher and Hamel, 1995; Laplante et al., 2005). Although the topographical projection patterns of BF nuclei are well-characterized, the functional organization and connectivity of neurons within the anatomical delimitations of BF nuclei are still debated. It is not known whether BF neurons are clustered relative to a precise function in their projection fields or if BF neurons are distributed randomly, acting independently in terms of function. Technical challenges have impeded clarification of the timing and localization of BF neuronal firing relative to external sensory stimuli. Some recent optogenetic studies are in the process of elucidating the firing patterns of BF neurons. For example, cholinergic neurons within the HDB and NBM were found to exhibit identical firing in mice performing a behavioral task, suggesting that BF neurons may constitute a unified broadcast system to the cortex (Hangya et al., 2015; Lin et al., 2015). Meanwhile, BF neurons projecting to primary auditory areas were found to be restricted to a small BF area distinct from the BF area projecting to secondary auditory areas (Chavez and Zaborszky, 2017).

The aim of the present study was to determine whether BF neurons that project to the visual cortex are finely organized and clustered retinotopically into subpopulations within the BF. Anatomical clustering would be consistent with neurons sharing a similar function. A broad distribution of cholinergic BF neurons projecting to different visual field representations would indicate complex BF organization. Thus, we performed restricted microinjections of cholera toxin fragment b (CTb) into multiple visual cortex sites according to the functional retinotopy proposed by Gias et al. (2005) and the architectonic organization described by Paxinos and Watson (1997). Labeled neurons within the BF were counted and their locations were identified in coronal brain sections. We expected that if there is topographic BF organization matching V1 retinotopy, then retrograde tracer placed in the visual cortex should result in the labeling of cells in a restricted part of the BF with a distribution pattern that is reproducible across animals.

## Materials and Methods

### Animal Preparation

Fifteen Long–Evans rats from Charles River Laboratories (Saint-Constant, QC, Canada), weighing 242–369 g, were used. All injections were unilateral; some animals received two tracer colors. Protocols were designed in accordance with the Canadian Council on Animal Care and approved by the Université de Montréal ethics committee (#14-164).

### Injections

We based our injection sites (*n* = 17) on Paxinos and Watson’s brain atlas coordinates (Paxinos and Watson, 1997), where V1m, V1b, V2m, and V2l are determined according to their architectonic organization. Animals were anesthetized with isoflurane (2–2.5%) and their temperatures were maintained at 37 ± 0.5°C by a thermostatic rectal temperature controller. Fluid replacement saline was given by subcutaneous bolus (1 cc/h). Each animal was placed in a stereotaxic apparatus (Kopf Instruments). The dorsal skin of the head was incised along the fronto-posterior axis, and then a burr hole was drilled through the skull above the visual cortex. A borosilicate micropipette linked to a 10-μl Hamilton syringe filled with mineral oil was used. CTb (1% in saline) conjugated with fluorescent Alexa 488 or 493 nm (Molecular probes) was loaded into micropipette tips. The filled micropipette was placed at the desired coordinates of cortical visual areas stereotaxically relative to Bregma’s suture; coordinates ranged antero-posteriorly from -5 to -9 mm and ranged laterally from 1 to 5 mm from the midline as shown in **Table 1**. CTb (0.5 μl) was injected at a rate of 0.1 μl/1 min by a syringe pump (Phd, Harvard apparatus) in the visual cortex. Dental acrylic was applied to secure the burr hole. The skin wound was sutured and each animal was returned to its home cage. Retrograde transport of the injected solution was allowed to proceed for 5–13 days.

**Table 1:** Distribution of retrogradely labeled cells by tracer injection site.

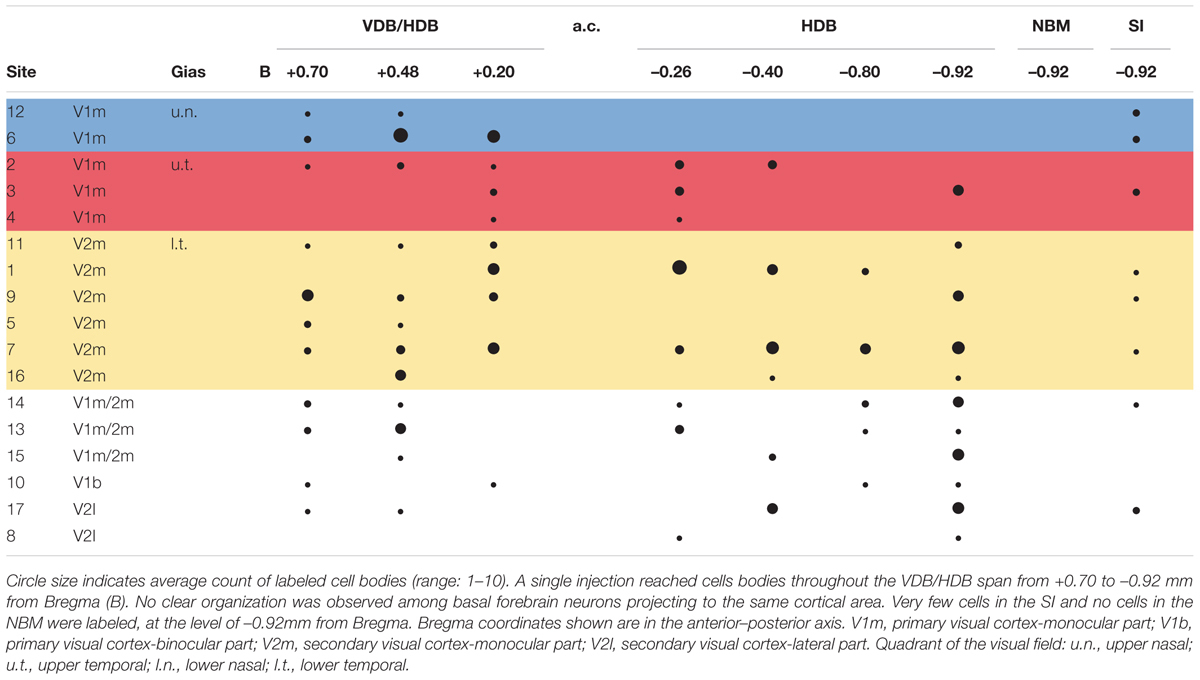

### Histochemistry

Animals were deeply anesthetized with pentobarbital then perfused with 4% paraformaldehyde and their brains were collected and cryoprotected in graded sucrose solutions (10–30%) for 3 days. Each brain was blocked with a matrix, flash frozen with isopentane, stored at -80°C, and then sliced into 50-μm coronal sections, proceeding from anterior to posterior areas, with a cryostat (Leica CM3050s). Sections were collected in series to preserve their order and stored in a glycerol preparation at 4°C before immunolabeling. Alternate CTb-tracer marked sections were labeled with choline acetyltransferase (ChAT). Briefly, sections were rinsed in phosphate buffer, blocked with 1.5% normal donkey serum (Jackson Immuno Research Laboratories, Inc., West Grove, PA, United States) for 90 min, immersed in goat anti-choline acetyltransferase 1:250 (Millipore, Ltd., Etobicoke, ON, Canada) for 24 h, and then incubated with Alexa Fluor anti-goat secondary antibody (1:200, Molecular Probes, Eugene, OR, United States) for 90 min. Finally, the sections were mounted on glass slide, and then cover slipped.

**FIGURE 1:**
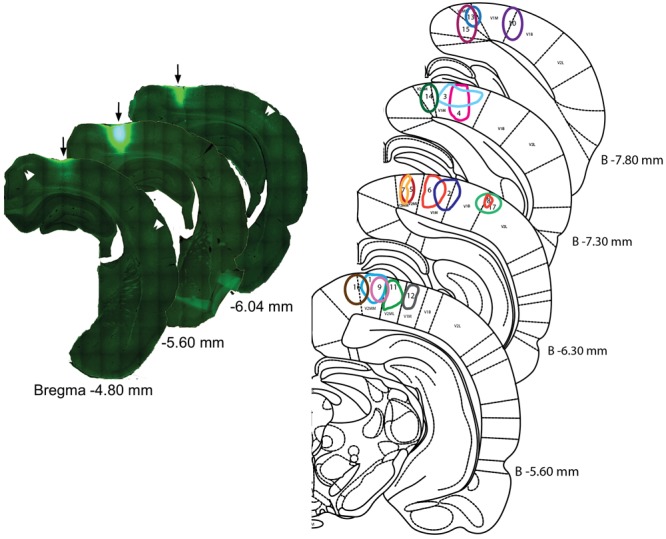
Injection sites in the visual areas. (Left) Example of a CTb injection into visual cortex. Reconstruction of the injection site #11 (arrows) with the aid of the virtual slices function in Stereoinvestigator software. The center section is the location of a 488 nm tracer-conjugated CTb injection; the reconstruction of other sections reveals the antero-posterior spread of the injection. Double arrow heads point to clusters of retrogradely stained neurons from adjacent cortical areas. Numbers indicate distance from Bregma. (Right) Representation of the different injection sites on coronal sections of the rat brain atlas. Edges of the injections site were drawn according to the saturation level of the CTb as recorded by the camera at the strongest fluorescence level. Distances from Bregma (in mm) are given for the different plates.

**FIGURE 2:**
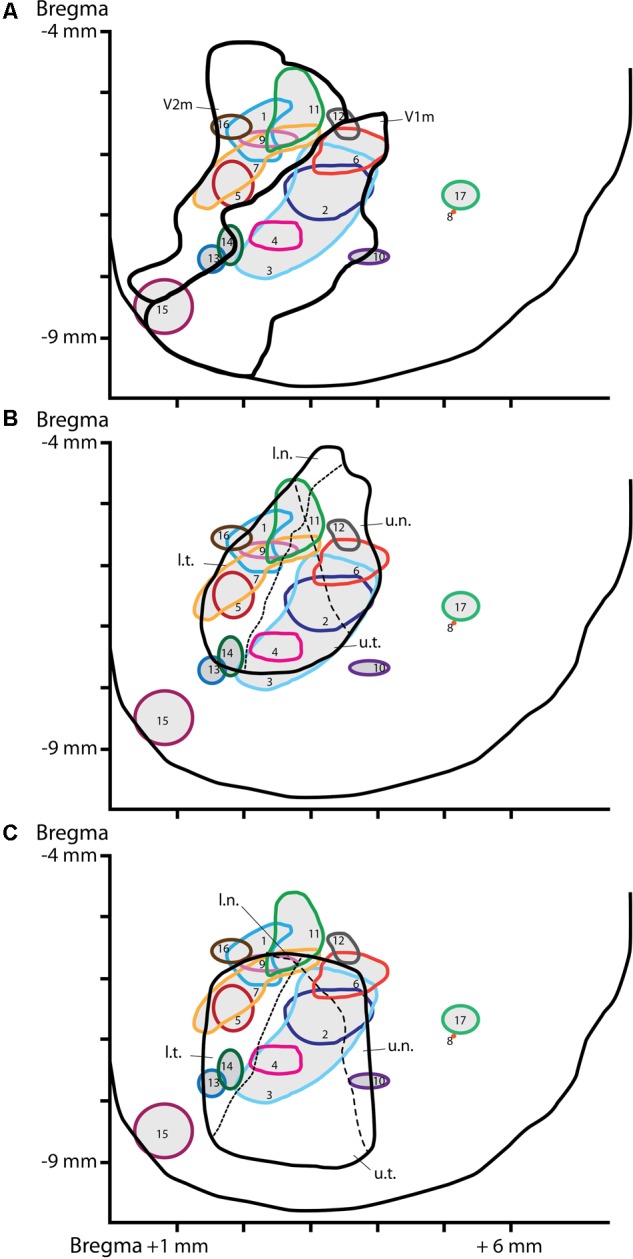
Location of the injection sites on a schematic dorsal view of the visual cortex. CTb injection sites reconstructed and plotted on a dorsal view of the visual cortex delineating visual areas according to Paxinos and Watson’s (1997) atlas **(A)**, the retinotopy from Gias et al. (2005) **(B)** or the retinotopy from Adams and Forrester (1968) **(C)**. The dashed lines represent the horizon (short dash) or the meridian (long dash) lines of the visual field. The x-axe depicts distance (in mm) from the mediolateral midline, the y-axe depict distance (in mm) from Bregma. V1m, primary visual cortex-monocular part; V2m, secondary visual cortex-monocular part. Quadrant of the visual field: u.n., upper nasal; u.t., upper temporal; l.n., lower nasal; l.t., lower temporal.

**FIGURE 3:**
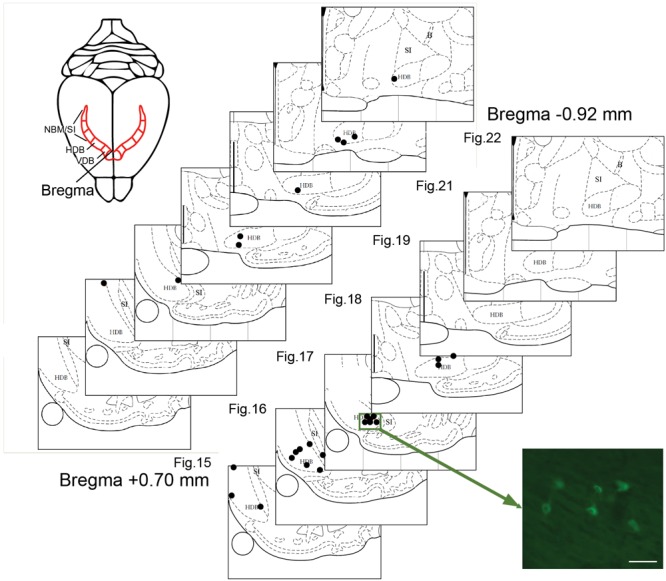
Plots of BF neurons marked with CTb injected in the visual cortex. Two representative cases of retrogradely labeled BF neurons (each dot represents one neuron) in V2m (site 11, upper panel) or V1m (site 6, lower panel). The locations are plotted on atlas plates (Figures 15–22; from +0.48 anterior to –0.92 mm posterior to Bregma). Most of the labeled cells are scattered in the HDB. The photomicrograph shows a cluster of fluorescent CTb-positive cells. Scale bar: 25 μm.

Tracer analysis was conducted by researchers who were blinded to the experimental conditions throughout the BF (from 1.5 anterior to 2 mm posterior to Bregma). Sections were selected according to a random start, and then sampled systematically (average spacing of 160 μm according to stereological recommendations) (Mouton et al., 2002). Marked cells were sparse (2.17 cells/section), which is not compatible with the use of a stereology grid. Therefore, we documented all encountered cells in each analyzed section. Limits of the sampled sections were delineated anatomically at low magnifications (2.5 and 10×) with reference to a rat brain atlas (Paxinos and Watson, 1997). Cell plotting was performed at higher magnification 63–100× magnification (Leica HCX PL Fluotar oil-immersion objective) under a Leica DMR microscope equipped with Stereoinvestigator software (v 9.13, Microbrightfields, Colchester, VT, United States) and a computer-driven motorized stage. To reconstruct injection sites, mosaic images were assembled (**Figure 1**) in Stereoinvestigator software linked to a camera (Retiga-200R fast 1394) and reconstructed on dorsal view of the visual cortex (**Figure 2**). Marked cells were plotted against Paxinos and Watson’s (1997) rat brain atlas plates (**Figure 3**). The HDB analysis range was +1.2 to -1.3 mm of Bregma, the SI analysis range was from -0.26 to -1.8 mm of Bregma. A confocal laser-scanning microscope (Leica TCS) was used to image double-labeled cells.

## Results

A mosaic reconstruction of the injection sites (**Figure 1**), visualized in coronal sections, was established to determine the spread of CTb injections within different quadrants of the visual cortex retinotopy. We reconstituted 17 injection sites from adjacent coronal sections located within V1m (monocular), or the monocular and lateral areas of secondary visual cortex (V2m and V2l) according to Paxinos and Watson’s (1997) atlas as shown in **Figure 2A**. These reconstituted sites were overlapped with the retinotopic maps established by Adams and Forrester (1968) and Gias et al. (2005) (**Figures 2B,C**) to establish the correspondence between BF neuronal organization and retinotopy.

Retrogradely labeled cells were sparse, independent of the extent or intensity of the corresponding injection site. At most, nine labeled BF cell bodies were detected within a single 50-μm-thick brain section. For example, a large injection site (#11) in rostral V2m was associated with a few retrogradely labeled cells that were widely distributed in the VDB/HDB or caudal HDB (**Figure 3** and **Table 1**). This example is representative of the results obtained with other injections leading to only one or two cells per section (0.66 cell/section). There were very few cell clusters. Of 17 injections sites, only four produced cell clusters in which ≥3 cells within 100 μm of one another (e.g., site #6, **Figure 3**). Cells were found throughout the anterior HDB to posterior HDB and SI, but none were located within the NBM at the level observed (-0.92 to 1.8 mm from Bregma). As expected, retrograde CTb tracer was observed in cell bodies, but not their dendrites, precluding visualization of interconnections between BF cells.

### Pattern of BF Neuronal Projections to Visual Areas

Six injections in V1m produced labeled cells in VDB, HDB, and SI regions. No clear pattern in the distribution of the cells among these BF regions was observed across injection sites in V1m (#2, #3, and #4), nor across injection sites in V2m (#1, #9, and #11) (**Figure 3**). A distribution pattern did not emerge across animals. Injections performed more laterally, within V1b or V2l, also resulted in widely distributed cells throughout the extent of the HDB and SI. However, looking at the data as a whole (**Table 1**), it became clear that neurons projecting to V1m were principally concentrated within the anterior HDB (+0.7 to -0.2 mm from Bregma), whereas neurons projecting to V2m were distributed throughout HDB (+0.7 to -0.9 mm from Bregma). Moreover, as shown clearly in **Table 1**, the anterior HDB (+0.7 to +0.48 mm from Bregma), as well as the SI, project to virtually all visual cortex subregions (V1m, V2m, and V2l).

### Correspondence With Retinotopic Maps

Puzzlingly, the functional retinotopic maps established by different authors differ slightly from one to another, depending on the mapping method (electrophysiology or optical imaging) and anesthesia used. We overlapped our injection site map with Gias’ retinotopy determined by optical imaging Gias et al. (2005) and Adams and Forrester (1968), who used pigmented rats. We did not compare our map with the retinotopy established by Espinoza and Thomas (1983) because their V1 map was substantially lateral compared to both previous maps and anatomical correlates. In Gias’ map, the upper visual field representation (u.t. and u.n.) corresponds to the architectonic structure of V1m and the lower field representation corresponds to V2m (l.t. and l.n.). Once again, there was no specific reproducible pattern of BF neuronal organization among the different rats. It is generally assumed that cells that function in synchrony are grouped into clusters. The cell clusters in BF observed in some rats were not redundant from the same quadrant. These results are not indicative of a predetermined organization of cholinergic projections to visual areas.

### Proportion of CTb Marked Cholinergic Cells

**FIGURE 4:**
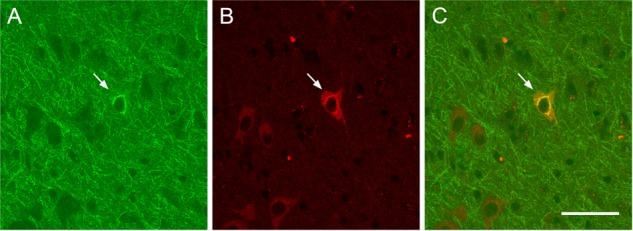
Examples of retrograde CTb-marked cholinergic cells. Confocal images showing a retrograde CTb-marked cell (**A**, arrow), ChAT-immunopositive cells (**B**, arrow), and merge of CTb and ChAT labels demonstrating a double labeled neuron (**C**, arrow). Scale bar: 50 μm.

As shown in **Figure 4**, ChAT immunolabeling revealed that the overwhelming majority (94.5%) of CTb retrogradely labeled cells were cholinergic, that is, they were double labeled for CTb and ChAT (**Figure 4**).

## Discussion

The present results show that BF neurons projecting to different parts of the visual cortex are intermingled within the VDB/HDB/SI regions. The only tendency observed was with respect to V1m receiving projections from the anterior HDB and SI, whereas V2m was observed to receive projections from neurons distributed at every level of the HDB/SI. Moreover, the SI and anterior HDB appear send projections to virtually all subregions of the visual cortex examined. However, we did not obtain evidence of BF neuron organization reproducing the retinotopic organization of the visual areas. These results suggest that any part of the VDB/HDB/anterior SI is likely to be able to modulate different areas of visual cortex.

### Spatial Organization of the Retrograde Labeled Cells

The present experiments revealed a low estimated number and broad dispersion of retrogradely labeled BF cells throughout the HDB/SI, suggesting that the axonal field of a single BF cell is quite restricted and that the axonal fields of different BF cells have quite limited, if any, overlap. A larger axonal field would have resulted in a greater density of labeled BF cells projecting directly or to the periphery of the injection site, even following a small injection. There is some controversy as to the size of the area of cortex innervated by individual BF neurons. In the rat, the terminal fields of cholinergic neurons appear to be restricted to a surface area that is ∼1.5 mm in diameter (Price and Stern, 1983; Saper, 1984), whereas more widespread termination has been reported in the cat (Adams et al., 1986). The present results tend to confirm a restricted axonal field of BF neuron projections to V1, a circumstance consistent with BF cholinergic axons containing few collateral fibers. Without excluding the possibility of *en passant* boutons underlying volume transmission, such restricted terminal fields also suggest that a single BF neuron has a rather precise projection. Meanwhile, the low number of cells found to have retrograde tracer from particular visual area injection sites suggests that BF projections to visual cortex are rather scattered. This circumstance, however, contrasts with the features of cholinergic innervation of occipital cortex, which shows a high density of cholinergic fibers (1.5–5.6 m per mm^2^ of cortical surface) and varicosities (0.6–2.3 × 10^6^ per mm^2^ of cortical surface). This density is much greater than that of any other neuromodulatory system (Mechawar et al., 2000). However, it is probable that *en passant* axons do not uptake microinjected CTb.

Our scarce observation of clusters further suggests limited overlap in axonal arborizations. High degrees of overlap would have been expected to result in more clusters. For example, a high density of labeled cells was seen in adjacent cortical regions in the present study (data not shown) and in thalamic nuclei previously (Conte et al., 2009). Clustering is a common hallmark of long-range cortico-cortical connections in the visual cortex of tree shrews, cats, monkeys and rats (Rockland and Lund, 1982; Livingstone and Hubel, 1984; Burkhalter, 1989; Gilbert and Wiesel, 1989). This type of organization appears when two cortical territories interact in a precise manner. The BF clusters shown in the present study appeared only following large injections, suggesting that the clustered cells project to distinct areas. With small injections, generally, only one cell or two cells were marked within single sections.

Notably, virtually all of the retrogradely labeled cells in this study were immunopositive for ChAT. This result is in agreement with other studies reporting that cortex-tracer receiving cells in the SI-NBM were largely also ChAT positive (80–90%) (Rye et al., 1984). Many studies on cortical projections have focused on the cholinergic component, while some have confirmed that there is a GABAergic component to the BF projections to the cerebral cortex (Fisher et al., 1988; Gritti et al., 1997). BF neurons can regulate the activity of cortical neurons not only via direct projections to the cortex, but also via indirect projections through the thalamus. The aim of the present study was not to establish the biochemical nature of the BF to the cortex connection, but we can confirm that most BF cells that are retrogradely labeled from visual cortex are cholinergic.

Together, these results indicate wide distributions of BF cells projecting to particular visual cortex sites and confirm a limited axonal arborization of cholinergic BF corticopetal projections. This arrangement fits with the BF’s apparent role in facilitating coordination between cortical subareas (Duque et al., 2000; Pinto et al., 2013). Our findings confirm a previous observation that projections to the prefrontal cortex or V1 originate from BF cells intermingled in the HDB (Laplante et al., 2005), and a recent study showing similar results in the auditory cortex (Chavez and Zaborszky, 2017).

### No Clear Recurrent Organization

Injections in the same area lead to different locations of stained BF cells from one animal to another, without evidence of a systematic organization pattern and certainly not reproducing the retinotopy of the visual cortex. If BF to V1 projections were precisely organized, one would expect that injection into a restricted quadrant of the visual cortex should lead to the consistent labeling of corresponding BF groups of cells and some pattern should repeat across animals. For example, in a tightly organized nucleus like the lateral geniculate, each part corresponds to a particular retinotopic region in the visual cortex (Reese and Jeffery, 1983; Piscopo et al., 2013; Castonguay et al., 2014). Previous studies have shown an organization that is relatively constant from one animal to the next, although there is a puzzling variability across published functional retinotopic maps (Adams and Forrester, 1968; Espinoza and Thomas, 1983; Gias et al., 2005). In particular, the medio-lateral coordinates of the visual cortex have been variable across studies, most likely due to the use of a flat mount versus a dorsal view of the brain. Our results suggest that a single locus within the BF is able to modulate diverse types of visual neurons over large surfaces of the visual cortex.

Nevertheless, there is a rostro-caudal organization tendency of HDB projections in which V1 is more abundantly innervated by rostral HDB than V2, which receives projections from all over the HDB. Such a rostro-caudal organization of projections is seen in NBM projections to the fronto-parietal cortices and has been well-characterized (Luiten et al., 1987; Gaykema et al., 1990). Likewise, BF cells projecting to primary auditory cortex were shown to be located in a more limited region than those projecting to secondary auditory cortex (Chavez and Zaborszky, 2017). These studies suggest that BF projections may play an integrative role in sensory cortices, which could be stronger in associative areas.

It can be assumed that distant projecting cells are activated by a larger variety of stimuli, thus increasing the integration weight of these projections. There is, however, a lack of understanding of how BF cells are activated, such as which region or stimulus elicits BF neuronal firing and if there is discrete BF activation of different BF subgroups. Many modulatory systems are able to activate cholinergic BF cells, such as the noradrenergic locus coeruleus, the serotoninergic raphe dorsalis, and the cholinergic pedonculopontine nucleus. The BF (especially the HDB) receives substantial innervation from the prefrontal cortex (Gaykema et al., 1991; Golmayo et al., 2003; Vertes, 2004) and is modulated by the amygdala and caudate putamen (Gielow and Zaborszky, 2017). It has been predicted that BF firing should be elicited by the prefrontal cortex (Golmayo et al., 2003), but this expectation has not yet been supported by anatomo-functional evidence of spontaneous activity. It is also not known whether the whole BF functions as a single entity, such that interconnections between BF subregions are able to either coordinate or isolate the cholinergic input affecting specific cortical areas. All these questions should be investigated to better understand how the BF modulates the cortex.

### Functional Effects of ACh Cells

Functional effects of BF cells are widespread, consistent with the neuromodulatory role of the BF. Single-unit recordings in the BF in combination with electroencephalographic (EEG) monitoring in animals during various behaviors have indicated that BF inputs to the neocortex correlate with activation of the neocortex (Pirch et al., 1986; Buzsaki et al., 1988; Metherate et al., 1992; Nunez, 1996; Duque et al., 2000). ACh-positive cells have been linked with frontal EEG state in the rat (Metherate et al., 1992) and EEG data correlate with the discharge profiles of NBM neurons (Buzsaki et al., 1988; Duque et al., 2000). This research supports the hypothesis that the cholinergic BF provides a steady background of neocortical activation that may facilitate or enhance the effects of other afferents to the neocortex (Duque et al., 2000). The stimulation of specific BF sites can produce either large-scale ACh release in the motor, somatosensory, and visual cortices, or restricted ACh release in one of these cortical areas (Jimenez-Capdeville et al., 1997). The present lack of evidence of clear retinotopic organization supports the view that the cholinergic BF has a broad modulatory role, reflected in EEG recordings, which can enhance local cortical processing.

This ubiquitous control of different visual areas by neighbor BF cholinergic neurons might also refine the transmission of visual fluxes throughout visual pathways. The visual processing results from coordinated actions of complementary visual areas with mirror functions (Zhuang et al., 2017). Thalamocortical inputs are processed within V1 but are also modulated by local, horizontal, as well as long-range recurrent microcircuits. Cholinergic modulation acts at every level of these microcircuits, enabling enhancement and facilitation of particular stimuli (Kang et al., 2014). For example, ACh might promote the co-activation of different cortical areas or layers (Obermayer et al., 2017) enabling selection of stimuli related to summation of the temporally coincident presynaptic spikes (Fries et al., 2002) or enabling expended cortical representation of sensory stimuli related to decorrelation between neurons (Goard and Dan, 2009). It can also trigger gamma oscillations allowing coordination between areas and triggering learning (Rodriguez et al., 2010; Nair et al., 2016).

## Conclusion

The present results indicate that BF projections to V1 and V2 are not organized according to the functional organization of the visual cortex. Rather, the results suggest that a single visual cortex region is controlled by different levels of the HDB and that the different BF subregions project broadly across visual cortex areas, although laminar projections were not investigated. Because ACh can facilitate or depress cortical responses, these findings imply that any BF region with projections to visual cortex can modulate visual processing at different hierarchical levels, an arrangement that confers a strong modulatory potency of cholinergic innervation upon visual cortex activities.

## Author Contributions

EV and FH-G: designed this study and drafted and wrote the manuscript. KJ and FH-G: contributed to the acquisition, analysis, and interpretation of the data in this study. EV: contributed to the interpretation of the data. All authors have approved the final version of the manuscript; agree to be accountable for all aspects of the work in ensuring that questions related to the accuracy or integrity of any part of the work are appropriately investigated and resolved; designated as authors qualify for authorship and who qualify for authorship are listed.

## Conflict of Interest Statement

The authors declare that the research was conducted in the absence of any commercial or financial relationships that could be construed as a potential conflict of interest.
